# 2-Chloro-1-(2,4,4-trimethyl-2,3,4,5-tetra­hydro-1*H*-1,5-benzodiazepin-1-yl)ethanone

**DOI:** 10.1107/S1600536813012324

**Published:** 2013-05-11

**Authors:** V. Thiruselvam, D. Deepa Rajakumari, A. Akila, S. Ponnuswamy, M. N. Ponnuswamy

**Affiliations:** a Centre of Advanced Study in Crystallography and Biophysics, University of Madras, Guindy Campus, Chennai 600 025, India; b Department of Chemistry, Government Arts College (Autonomous), Coimbatore 641 018, India

## Abstract

In the title compound, C_14_H_19_ClN_2_O, the diazepine ring adopts a boat conformation. The Cl atom of the chloro­acetyl group is *trans* to the N atom of the diazepine ring. In the crystal, the mol­ecules form chains running along the diagonal of the *ac* plane through N—H⋯O hydrogen bonds.

## Related literature

For the biological activity of benzodiazepine derivatives, see: Ponnuswamy *et al.* (2006 ▶); Rahbaek *et al.* (1999 ▶). For related structures see: Thiruvalluvar & Ponnuswamy (2007 ▶); Kavitha *et al.* (2012 ▶). For puckering parameters, see: Cremer & Pople (1975 ▶) and for asymmetry parameters, see: Nardelli (1983 ▶). For bond-length data, see: Allen *et al.* (1987 ▶).
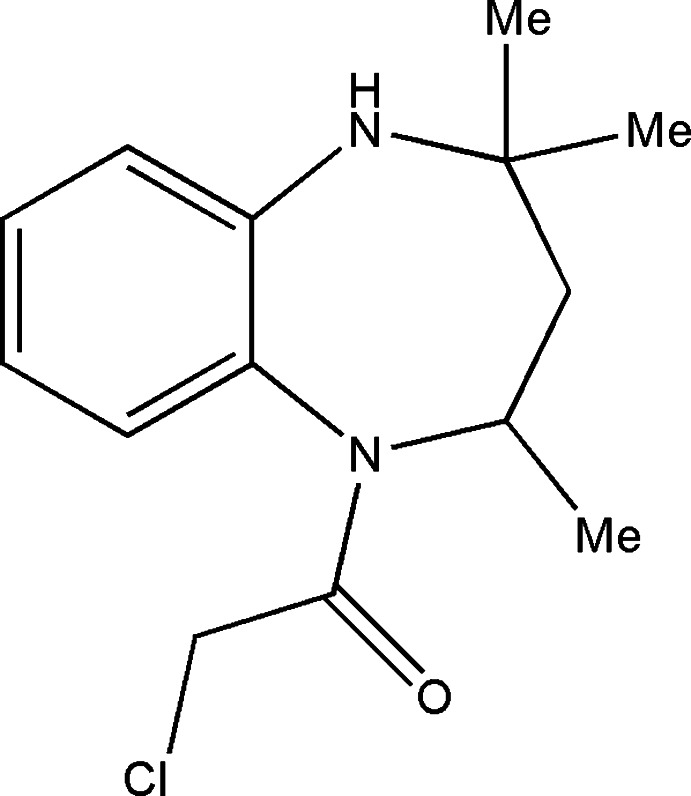


## Experimental

### 

#### Crystal data

C_14_H_19_ClN_2_O
*M* *_r_* = 266.76
Monoclinic, 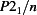

*a* = 10.3971 (3) Å
*b* = 12.2589 (3) Å
*c* = 11.0994 (3) Å
β = 93.953 (1)°
*V* = 1411.33 (7) Å^3^
*Z* = 4
Mo *K*α radiation
μ = 0.26 mm^−1^
*T* = 293 K
0.23 × 0.22 × 0.20 mm

#### Data collection

Bruker SMART APEXII CCD diffractometer
Absorption correction: multi-scan (*SADABS*; Bruker, 2008 ▶) *T*_min_ = 0.942, *T*_max_ = 0.949
13339 measured reflections
3568 independent reflections
2767 reflections with *I* > 2σ(*I*)
*R*_int_ = 0.018

#### Refinement

*R*[*F* ^2^ > 2σ(*F* ^2^)] = 0.040
*wR*(*F* ^2^) = 0.120
*S* = 1.07
3568 reflections
167 parameters
H atoms treated by a mixture of independent and constrained refinement
Δρ_max_ = 0.22 e Å^−3^
Δρ_min_ = −0.27 e Å^−3^

### 

Data collection: *APEX2* (Bruker, 2008 ▶); cell refinement: *SAINT* (Bruker, 2008 ▶); data reduction: *SAINT*; program(s) used to solve structure: *SHELXS97* (Sheldrick, 2008 ▶); program(s) used to refine structure: *SHELXL97* (Sheldrick, 2008 ▶); molecular graphics: *ORTEP-3 for Windows* (Farrugia, 2012 ▶); software used to prepare material for publication: *SHELXL97* and *PLATON* (Spek, 2009 ▶).

## Supplementary Material

Crystal structure: contains datablock(s) global, I. DOI: 10.1107/S1600536813012324/bt6901sup1.cif

Structure factors: contains datablock(s) I. DOI: 10.1107/S1600536813012324/bt6901Isup2.hkl

Supplementary material file. DOI: 10.1107/S1600536813012324/bt6901Isup3.cml

Additional supplementary materials:  crystallographic information; 3D view; checkCIF report

## Figures and Tables

**Table 1:** Hydrogen-bond geometry (Å, °)

*D*—H⋯*A*	*D*—H	H⋯*A*	*D*⋯*A*	*D*—H⋯*A*
N1—H1⋯O1^i^	0.835 (19)	2.276 (19)	3.1049 (16)	171.5 (17)

Symmetry code: (i) 
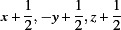
.

**Fig. 1.**
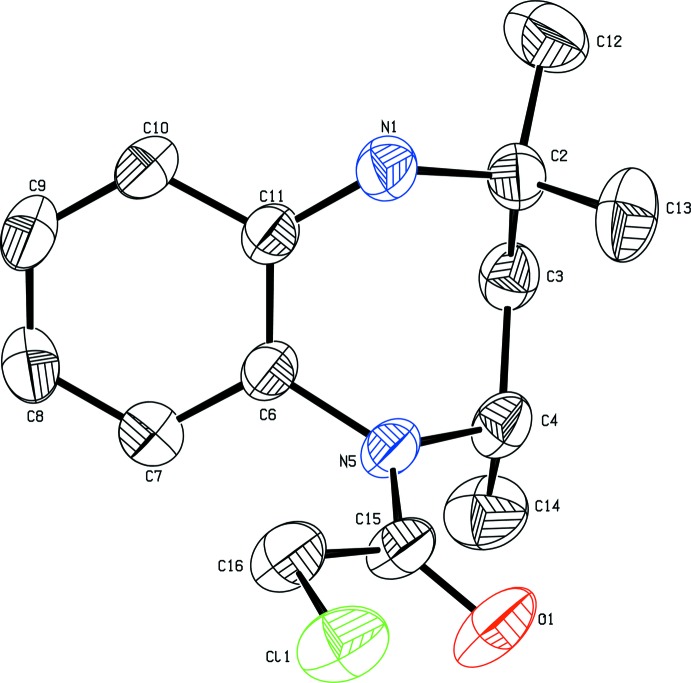
The molecular structure of the title compound, showing the atomic numbering and displacement ellipsoids drawn at 50% probability level.

**Fig. 2.**
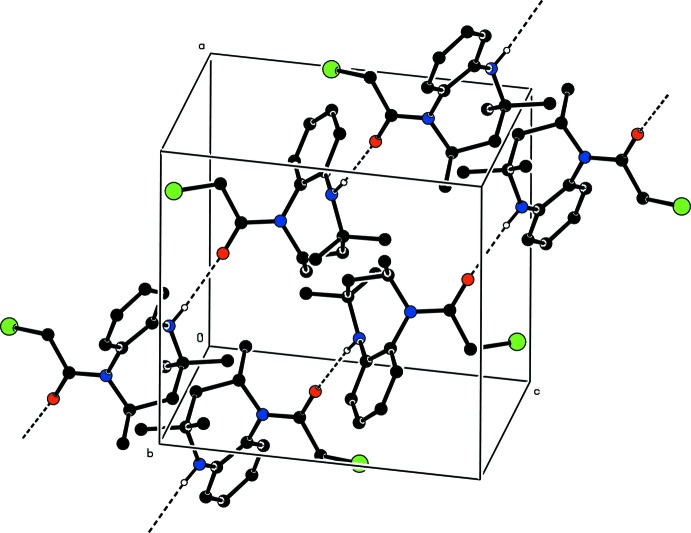
The crystal packing of the molecules. H atoms not involved in hydrogen bonding (dashed lines) have been omitted for clarity.

## supplementary crystallographic information

### Comment

Various substituted 1,5-benzodiazepines have been synthesized and their stereochemistry has been reported (Ponnuswamy *et al.*, 2006). Among these, the benzodiazepines act as a class of psychoactive drugs. Benzodiazepines are known for their natural occurrence in filamentous fungi and actinomycetes of the genera pencillium, aspergillus and streptomyces (Rahbaek *et al.*, 1999). Benzodiazepines from aspergillus include asperlicin, which is used for the treatment of gastrointestinal and central nervous system disorders. Against this background and to ascertain the molecular structure and conformation, the X-ray crystal structure determination of the title compound has been carried out.

The *ORTEP* plot of the molecule is shown in Fig. 1. The chloro substituted benzodiazepine derivative crystallizes in the monoclinic space group *P*2_1_/*n*. The diazepine ring system adopts a boat conformation (Kavitha *et al.*, 2012). The puckering parameters (Cremer & Pople, 1975) and the asymmetry parameters (Nardelli, 1983) are: q_2_=0.9558 (2) Å, q_3_ = 0.1431 (2) Å, φ_2_ = 16.93 (9)° and Δ_s_(C6)= 25.13 (2)°. The Cl atom of the chloroacetyl group is trans to the N atom of the diazepine ring which is evidenced from the torsion angle [N5—C15—C16—CL1=]-157.5 (1)°. The bond lengths C16—CL1 and C15—O1 [1.770 (2) Å & 1.219 (2) Å] are comparable with the mean value reported in the literature (Allen *et al.*, 1987). The carbonyl group is oriented *anti* to C6 [C6—N5—C15—O1=] 169.7 (2)° and *syn* to C4 [C4—N5—C15—O1=] -5.12 (2)°. The crystal packing shows that the molecules form linear chains linked through N—H···O hydrogen bonds. The chains run along the diagonal of the ac plane (Fig. 2).

### Experimental

To an ice cold solution of tetrahydrobenzodiazepine (1.9 g, 10 m.mol) in anhydrous benzene (50 ml), triethylamine (4 ml, 30 m.mol) and chloroacetylchloride (2.4 ml, 30 m.mol) were added and stirred at room temperature. The resulting solid was purified by recrystallization from benzene to yield pale yellow crystals.

### Refinement

The H atom bonded to N was freely refined. C-bound H atoms were positioned geometrically (C–H = 0.93–0.98 Å) and allowed to ride on their parent atoms, with *U*_iso_(H) = 1.5*U*_eq_(C) for methyl H atoms and 1.2*U*_eq_(C) for all other H atoms.

### Crystal data

**Table:**

C_14_H_19_ClN_2_O	*F*(000) = 568
*M**_r_* = 266.76	*D*_x_ = 1.255 Mg m^−^^3^
Monoclinic, *P*2_1_/*n*	Mo *K*α radiation, λ = 0.71073 Å
Hall symbol: -P 2yn	Cell parameters from 3568 reflections
*a* = 10.3971 (3) Å	θ = 2.5–28.5°
*b* = 12.2589 (3) Å	µ = 0.26 mm^−^^1^
*c* = 11.0994 (3) Å	*T* = 293 K
β = 93.953 (1)°	Block, pale yellow
*V* = 1411.33 (7) Å^3^	0.23 × 0.22 × 0.20 mm
*Z* = 4	

### Data collection

**Table:**

Bruker SMART APEXII CCD diffractometer	3568 independent reflections
Radiation source: fine-focus sealed tube	2767 reflections with *I* > 2σ(*I*)
Graphite monochromator	*R*_int_ = 0.018
ω and φ scans	θ_max_ = 28.5°, θ_min_ = 2.5°
Absorption correction: multi-scan (*SADABS*; Bruker, 2008)	*h* = −11→13
*T*_min_ = 0.942, *T*_max_ = 0.949	*k* = −16→14
13339 measured reflections	*l* = −14→14

### Refinement

**Table:**

Refinement on *F*^2^	Primary atom site location: structure-invariant direct methods
Least-squares matrix: full	Secondary atom site location: difference Fourier map
*R*[*F*^2^ > 2σ(*F*^2^)] = 0.040	Hydrogen site location: inferred from neighbouring sites
*wR*(*F*^2^) = 0.120	H atoms treated by a mixture of independent and constrained refinement
*S* = 1.07	*w* = 1/[σ^2^(*F*_o_^2^) + (0.0584*P*)^2^ + 0.253*P*] where *P* = (*F*_o_^2^ + 2*F*_c_^2^)/3
3568 reflections	(Δ/σ)_max_ < 0.001
167 parameters	Δρ_max_ = 0.22 e Å^−^^3^
0 restraints	Δρ_min_ = −0.27 e Å^−^^3^

### Special details

**Table:**

Geometry. All e.s.d.'s (except the e.s.d. in the dihedral angle between two l.s. planes) are estimated using the full covariance matrix. The cell e.s.d.'s are taken into account individually in the estimation of e.s.d.'s in distances, angles and torsion angles; correlations between e.s.d.'s in cell parameters are only used when they are defined by crystal symmetry. An approximate (isotropic) treatment of cell e.s.d.'s is used for estimating e.s.d.'s involving l.s. planes.
Refinement. Refinement of *F*^2^ against ALL reflections. The weighted *R*-factor *wR* and goodness of fit *S* are based on *F*^2^, conventional *R*-factors *R* are based on *F*, with *F* set to zero for negative *F*^2^. The threshold expression of *F*^2^ > σ(*F*^2^) is used only for calculating *R*-factors(gt) *etc*. and is not relevant to the choice of reflections for refinement. *R*-factors based on *F*^2^ are statistically about twice as large as those based on *F*, and *R*- factors based on ALL data will be even larger.

### Fractional atomic coordinates and isotropic or equivalent isotropic displacement parameters (Å^2^)

**Table:**

	*x*	*y*	*z*	*U*_iso_*/*U*_eq_	
C2	0.51697 (13)	0.25960 (11)	0.45502 (13)	0.0430 (3)	
C3	0.42704 (13)	0.16143 (11)	0.43872 (12)	0.0418 (3)	
H3A	0.4703	0.0989	0.4767	0.050*	
H3B	0.3511	0.1759	0.4822	0.050*	
C4	0.38266 (12)	0.12997 (12)	0.31006 (12)	0.0421 (3)	
H4	0.3297	0.1897	0.2751	0.051*	
C6	0.61213 (12)	0.07242 (11)	0.29286 (11)	0.0378 (3)	
C7	0.65316 (15)	−0.02988 (12)	0.25761 (13)	0.0472 (3)	
H7	0.5998	−0.0720	0.2055	0.057*	
C8	0.77192 (16)	−0.06946 (13)	0.29912 (15)	0.0557 (4)	
H8	0.7995	−0.1377	0.2749	0.067*	
C9	0.84918 (16)	−0.00669 (15)	0.37691 (16)	0.0584 (4)	
H9	0.9311	−0.0314	0.4026	0.070*	
C10	0.80708 (14)	0.09221 (13)	0.41727 (13)	0.0490 (3)	
H10	0.8600	0.1321	0.4720	0.059*	
C11	0.68638 (12)	0.13399 (11)	0.37769 (11)	0.0373 (3)	
C12	0.5320 (2)	0.28786 (16)	0.58975 (16)	0.0687 (5)	
H12A	0.5650	0.2257	0.6344	0.103*	
H12B	0.4496	0.3078	0.6171	0.103*	
H12C	0.5908	0.3478	0.6021	0.103*	
C13	0.46332 (16)	0.35866 (13)	0.38470 (19)	0.0635 (5)	
H13A	0.5217	0.4189	0.3970	0.095*	
H13B	0.3810	0.3780	0.4127	0.095*	
H13C	0.4535	0.3413	0.3002	0.095*	
C14	0.30135 (17)	0.02757 (15)	0.30537 (17)	0.0618 (4)	
H14A	0.2749	0.0108	0.2229	0.093*	
H14B	0.2265	0.0389	0.3499	0.093*	
H14C	0.3510	−0.0319	0.3404	0.093*	
C15	0.48304 (13)	0.14054 (13)	0.11892 (12)	0.0449 (3)	
C16	0.60674 (15)	0.13616 (16)	0.05310 (14)	0.0554 (4)	
H16A	0.6253	0.0611	0.0327	0.067*	
H16B	0.6780	0.1633	0.1057	0.067*	
N1	0.64710 (12)	0.23518 (10)	0.41521 (12)	0.0446 (3)	
H1	0.7043 (18)	0.2675 (14)	0.4580 (16)	0.057 (5)*	
N5	0.49529 (10)	0.11740 (9)	0.23764 (9)	0.0394 (3)	
O1	0.38003 (10)	0.16453 (13)	0.06665 (10)	0.0683 (4)	
Cl1	0.59194 (4)	0.21546 (4)	−0.07992 (4)	0.06677 (17)	

### Atomic displacement parameters (Å^2^)

**Table:**

	*U*^11^	*U*^22^	*U*^33^	*U*^12^	*U*^13^	*U*^23^
C2	0.0408 (7)	0.0429 (7)	0.0454 (7)	0.0064 (6)	0.0046 (6)	−0.0003 (6)
C3	0.0388 (7)	0.0465 (7)	0.0405 (7)	0.0047 (6)	0.0059 (5)	0.0050 (5)
C4	0.0304 (6)	0.0524 (8)	0.0436 (7)	0.0024 (6)	0.0021 (5)	0.0048 (6)
C6	0.0312 (6)	0.0460 (7)	0.0359 (6)	0.0047 (5)	−0.0011 (5)	0.0016 (5)
C7	0.0464 (8)	0.0492 (8)	0.0458 (7)	0.0031 (6)	0.0013 (6)	−0.0063 (6)
C8	0.0541 (9)	0.0532 (8)	0.0599 (9)	0.0185 (7)	0.0053 (7)	−0.0055 (7)
C9	0.0413 (8)	0.0722 (10)	0.0605 (9)	0.0220 (7)	−0.0050 (7)	−0.0028 (8)
C10	0.0371 (7)	0.0616 (9)	0.0467 (7)	0.0073 (6)	−0.0082 (6)	−0.0028 (6)
C11	0.0330 (6)	0.0436 (7)	0.0350 (6)	0.0037 (5)	0.0009 (5)	0.0029 (5)
C12	0.0760 (12)	0.0747 (12)	0.0563 (10)	−0.0039 (9)	0.0107 (9)	−0.0193 (8)
C13	0.0508 (9)	0.0459 (9)	0.0940 (13)	0.0107 (7)	0.0068 (9)	0.0146 (8)
C14	0.0490 (9)	0.0716 (11)	0.0649 (10)	−0.0161 (8)	0.0047 (7)	−0.0021 (8)
C15	0.0334 (7)	0.0613 (9)	0.0393 (7)	−0.0025 (6)	−0.0036 (5)	0.0043 (6)
C16	0.0420 (8)	0.0808 (11)	0.0434 (7)	−0.0005 (7)	0.0018 (6)	0.0056 (7)
N1	0.0350 (6)	0.0442 (6)	0.0537 (7)	0.0002 (5)	−0.0026 (5)	−0.0073 (5)
N5	0.0294 (5)	0.0513 (6)	0.0369 (5)	0.0030 (5)	−0.0025 (4)	0.0014 (5)
O1	0.0380 (6)	0.1162 (10)	0.0492 (6)	0.0051 (6)	−0.0072 (5)	0.0230 (6)
Cl1	0.0581 (3)	0.0963 (4)	0.0461 (2)	−0.0130 (2)	0.00504 (18)	0.01223 (19)

### Geometric parameters (Å, º)

**Table:**

C2—N1	1.4825 (18)	C10—C11	1.3979 (18)
C2—C3	1.527 (2)	C10—H10	0.9300
C2—C13	1.528 (2)	C11—N1	1.3793 (17)
C2—C12	1.532 (2)	C12—H12A	0.9600
C3—C4	1.5199 (19)	C12—H12B	0.9600
C3—H3A	0.9700	C12—H12C	0.9600
C3—H3B	0.9700	C13—H13A	0.9600
C4—N5	1.4734 (16)	C13—H13B	0.9600
C4—C14	1.512 (2)	C13—H13C	0.9600
C4—H4	0.9800	C14—H14A	0.9600
C6—C7	1.3896 (19)	C14—H14B	0.9600
C6—C11	1.3971 (18)	C14—H14C	0.9600
C6—N5	1.4327 (16)	C15—O1	1.2184 (17)
C7—C8	1.376 (2)	C15—N5	1.3453 (17)
C7—H7	0.9300	C15—C16	1.523 (2)
C8—C9	1.374 (2)	C16—Cl1	1.7656 (16)
C8—H8	0.9300	C16—H16A	0.9700
C9—C10	1.374 (2)	C16—H16B	0.9700
C9—H9	0.9300	N1—H1	0.835 (19)
			
N1—C2—C3	111.68 (11)	C6—C11—C10	117.15 (12)
N1—C2—C13	108.47 (12)	C2—C12—H12A	109.5
C3—C2—C13	111.52 (12)	C2—C12—H12B	109.5
N1—C2—C12	107.62 (13)	H12A—C12—H12B	109.5
C3—C2—C12	108.31 (12)	C2—C12—H12C	109.5
C13—C2—C12	109.14 (14)	H12A—C12—H12C	109.5
C4—C3—C2	117.06 (11)	H12B—C12—H12C	109.5
C4—C3—H3A	108.0	C2—C13—H13A	109.5
C2—C3—H3A	108.0	C2—C13—H13B	109.5
C4—C3—H3B	108.0	H13A—C13—H13B	109.5
C2—C3—H3B	108.0	C2—C13—H13C	109.5
H3A—C3—H3B	107.3	H13A—C13—H13C	109.5
N5—C4—C14	110.96 (12)	H13B—C13—H13C	109.5
N5—C4—C3	109.75 (10)	C4—C14—H14A	109.5
C14—C4—C3	112.07 (12)	C4—C14—H14B	109.5
N5—C4—H4	108.0	H14A—C14—H14B	109.5
C14—C4—H4	108.0	C4—C14—H14C	109.5
C3—C4—H4	108.0	H14A—C14—H14C	109.5
C7—C6—C11	120.75 (12)	H14B—C14—H14C	109.5
C7—C6—N5	119.64 (12)	O1—C15—N5	122.55 (13)
C11—C6—N5	119.56 (12)	O1—C15—C16	121.58 (13)
C8—C7—C6	120.64 (14)	N5—C15—C16	115.87 (12)
C8—C7—H7	119.7	C15—C16—Cl1	110.72 (11)
C6—C7—H7	119.7	C15—C16—H16A	109.5
C9—C8—C7	119.02 (14)	Cl1—C16—H16A	109.5
C9—C8—H8	120.5	C15—C16—H16B	109.5
C7—C8—H8	120.5	Cl1—C16—H16B	109.5
C8—C9—C10	120.89 (14)	H16A—C16—H16B	108.1
C8—C9—H9	119.6	C11—N1—C2	124.54 (12)
C10—C9—H9	119.6	C11—N1—H1	112.6 (12)
C9—C10—C11	121.33 (14)	C2—N1—H1	111.3 (12)
C9—C10—H10	119.3	C15—N5—C6	121.36 (11)
C11—C10—H10	119.3	C15—N5—C4	119.16 (11)
N1—C11—C6	121.87 (12)	C6—N5—C4	119.29 (10)
N1—C11—C10	120.84 (12)		
			
N1—C2—C3—C4	−70.58 (15)	C6—C11—N1—C2	48.60 (19)
C13—C2—C3—C4	50.97 (17)	C10—C11—N1—C2	−135.87 (15)
C12—C2—C3—C4	171.08 (13)	C3—C2—N1—C11	−5.18 (19)
C2—C3—C4—N5	52.53 (16)	C13—C2—N1—C11	−128.48 (15)
C2—C3—C4—C14	176.30 (12)	C12—C2—N1—C11	113.57 (15)
C11—C6—C7—C8	−4.6 (2)	O1—C15—N5—C6	169.69 (15)
N5—C6—C7—C8	172.60 (14)	C16—C15—N5—C6	−10.9 (2)
C6—C7—C8—C9	0.5 (2)	O1—C15—N5—C4	−5.1 (2)
C7—C8—C9—C10	2.8 (3)	C16—C15—N5—C4	174.26 (13)
C8—C9—C10—C11	−2.1 (3)	C7—C6—N5—C15	−63.37 (18)
C7—C6—C11—N1	−179.24 (13)	C11—C6—N5—C15	113.83 (15)
N5—C6—C11—N1	3.60 (19)	C7—C6—N5—C4	111.44 (15)
C7—C6—C11—C10	5.1 (2)	C11—C6—N5—C4	−71.37 (16)
N5—C6—C11—C10	−172.09 (12)	C14—C4—N5—C15	86.63 (16)
C9—C10—C11—N1	−177.52 (15)	C3—C4—N5—C15	−148.96 (12)
C9—C10—C11—C6	−1.8 (2)	C14—C4—N5—C6	−88.29 (15)
O1—C15—C16—Cl1	21.9 (2)	C3—C4—N5—C6	36.11 (17)
N5—C15—C16—Cl1	−157.53 (12)		

### Hydrogen-bond geometry (Å, º)

**Table:**

*D*—H···*A*	*D*—H	H···*A*	*D*···*A*	*D*—H···*A*
N1—H1···O1^i^	0.835 (19)	2.276 (19)	3.1049 (16)	171.5 (17)

Symmetry code: (i) *x*+1/2, −*y*+1/2, *z*+1/2.
